# High-efficiency generation of bi-functional holography with metasurfaces

**DOI:** 10.1515/nanoph-2024-0677

**Published:** 2025-02-20

**Authors:** Changhong Dai, Tong Liu, Dongyi Wang, Lei Zhou

**Affiliations:** State Key Laboratory of Surface Physics, Key Laboratory of Micro and Nano Photonic Structures (Ministry of Education), Shanghai Key Laboratory of Metasurfaces for Light Manipulation and Department of Physics, Fudan University, Shanghai 200438, China; Department of Physics, The Hong Kong University of Science and Technology, Clear Water Bay, Kowloon, Hong Kong, China; Department of Physics, The University of Hong Kong, Hong Kong 999077, China; Shanghai Key Laboratory of Metasurfaces for Light Manipulation, Fudan University, Shanghai, 200433, China; State Key Laboratory of Surface Physics, Key Laboratory of Micro and Nano Photonic Structures (Ministry of Education) and Department of Physics, Fudan University, 200433, Shanghai, People’s Republic of China

**Keywords:** bi-functional holography, ultra-thin, high-efficiency, meta-hologram

## Abstract

Holography is a highly desired technology in modern photonics, yet setups for traditional generating methods suffer from complexity and bulky sizes. While metasurface-based holography exhibits advantages of compactness and easy-fabrication, most meta-holograms realized so far exhibit only single functionality, with a few multifunctional ones suffering from imbalances of efficiency and device-thickness. Here, we propose a generic approach to design *ultra-thin* metasurfaces for realization of multiple holographic images with *high efficiencies*, and experimentally verify the concept in the telecom regime. We first design a series of high-efficiency reflective meta-atoms exhibiting incident-spin-delinked reflection phases governed by geometric and resonant mechanisms, and experimentally characterize their optical properties at wavelengths around 1,064 nm. We next experimentally demonstrate a single-functional meta-hologram as a benchmark test. Finally, we employ the designed meta-atoms to construct a metasurface with the thickness ∼1/4*λ*, and experimentally demonstrate its capability of generating two distinct holographic images under illuminations of circularly polarized light beams with different helicities, possessing generation efficiencies ∼48.08 %. Our work provides a highly-efficient and ultra-compact platform to generate multifunctional holographic images, which may inspire numerous applications in integration optics.

## 1 Introduction

Holography, an advanced optical technology for recording and reconstructing light fields, has garnered increasing interests due to its broad applications in modern optics, such as imaging, displays, data storage, and microscopy [1], [2]. However, traditional holography technology requires complex setups to generate holograms, exhibiting several-wavelengths’ thicknesses. These inherent limitations pose obstacles for the holography technology from being integrated into on-chip photonics applications.

Metasurfaces, ultra-thin metamaterials composed of subwavelength microstructures (i.e., meta-atoms) arranged in specific sequences, provide an alternative and powerful platform for manipulating light. Based on metasurfaces constructed by meta-atoms exhibiting tailored local light-scattering properties (including transmission/reflection amplitudes and phases), numerous intriguing light-manipulation effects have been demonstrated, such as polarization control [3], [4], [5], [6], anomalous light bending [7], [8], flat lenses [9], [10], [11], [12], surface wave excitation [13], [14] and many others [15], [16], [17]. Recently, based on reflection/transmission phase distributions retrieved by the Gerchberg–Saxton (GS) algorithm, metasurfaces have been constructed and employed to generate target far-field (FF) holographic images [18], [19], [20]. However, meta-holograms realized in earlier years [21], [22], [23], though being ultra-thin in thickness, can only generate one single holographic image. While many efforts have been devoted to building multifunctional metasurfaces for generation of multiple images upon light illuminations with different polarizations or wavelengths, meta-holograms realized so far still suffer from imbalances between device thickness and working efficiency/performance. For example, bi-functional meta-holograms [24], [25] constructed by merging two different Pancharatnam–Berry (PB) metasurfaces can be ultra-thin, but suffer from background-scattering noises which may degrade the working efficiency and imaging quality. Moreover, employing PB meta-atoms exhibiting geometric phases only, the meta-hologram realized in Ref. [25] is not a real bi-functional device, as switching the helicity of the incident circularly polarized light does not generate a new holographic pattern, but rather a centrosymmetric-transformation of the pattern realized previously. While all-dielectric bi-functional meta-holograms [26], [27] can realize high-resolution holographic images with high efficiencies, they are unfortunately of wavelength-scale thicknesses, caused by insufficient capabilities of dielectric resonators to control light at the deep-subwavelength scales.

Here, we propose a generic approach to design *ultra-thin* bi-functional meta-holograms to realize two distinct holographic images with *high efficiencies*. We first design a series of meta-atoms in metal/insulator/metal (MIM) configuration exhibiting reflection phases governed by both resonant and geometric mechanisms, and then utilize them to construct bi-functional meta-holograms based on phase distributions retrieved from two pre-designed holographic images. In particular, employing ultra-thin composite MIM meta-atoms exhibiting both PB and resonant phases, we can overcome the longstanding issue of imbalance between device thickness and efficiency in previous works on bi-functional meta-holography. We experimentally demonstrate two sets of meta-holograms, one being a single-functional case as a benchmark test and the other set being bi-functional ones (three different bi-functional meta-devices), to verify our concept in the telecom wavelength regime. Our meta-devices are of 1/4*λ* thicknesses, yet exhibit experimental efficiencies exceeding 30 %. Compared to dielectric resonators, our MIM meta-atoms utilize *lateral resonances* to modulate reflection phases with high reflectance, which can thus be deep-subwavelength. By addressing the imbalance between device thickness and efficiency, our results provide an alternative meta-platform to manipulate light with multi-functionalities, which may inspire many applications in integration optics.

## 2 Results

### 2.1 Generic design strategy

We now establish a generic strategy to design a metasurface, which, as shined by circularly polarized (CP) light beams with different helicities, can generate two distinct pre-designed holographic images in the FF. Suppose that our target holographic images are characterized by two momentum-space intensity distributions $I_{\text{tar,}\left|+\right\rangle }^{h}\left(\vec{k}\right)$ and $I_{\text{tar,}\left|-\right\rangle }^{h}\left(\vec{k}\right)$ , corresponding to the spatial field distributions obtained on the imaging plane of a lens used experimentally to record the images. We can use the GS algorithm to retrieve the phase distributions $\Phi_{\left|+\right\rangle }^{m}\left(\vec{r}\right)$ and $\Phi_{\left|-\right\rangle }^{m}\left(\vec{r}\right)$ of two planar sources that can generate these two holographic images. To realize these two images using a single meta-device, we need to design a single metasurface to exhibit reflection-phase distributions $\Phi_{\left|+\right\rangle }^{m}\left(\vec{r}\right)$ and $\Phi_{\left|-\right\rangle }^{m}\left(\vec{r}\right)$ , respectively, as illuminated by LCP and RCP light beams. To construct such a metasurface, our first task is to design a set of meta-atoms exhibiting spin-delinked reflection phases under illuminations of CP light beams of different helicities.

**Figure 1:**
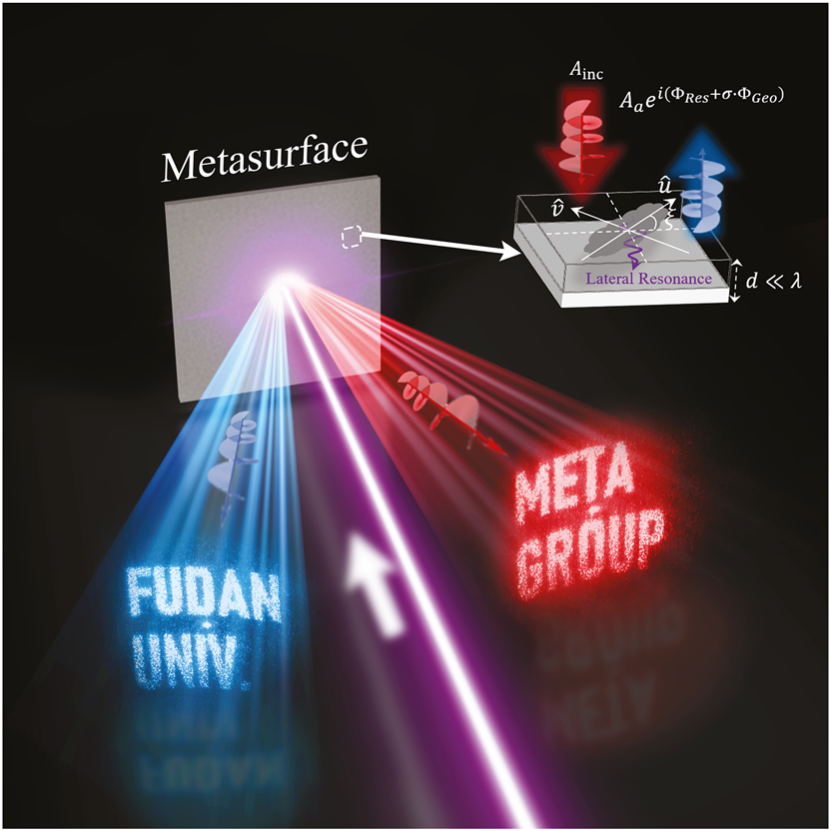
Schematics of bi-functional holography based on metasurfaces. Constructing metasurfaces with ultra-thin meta-atoms exhibiting reflection phases of both resonant and geometric origins (see the inset), we can generate two distinct pre-designed holographic images via shining the metasurfaces with circularly polarized light with different helicities.

As schematically illustrated in the inset of Figure 1, our meta-atoms are in metal/insulator/metal (MIM) configurations, each consisting of an anisotropic cross-shaped metallic resonator rotated by an angle of *ξ* and a continuous metal film, separated by a dielectric spacer. The continuous metal film ensures that our meta-atoms allow no light-transmission, thus we only need to study their reflection properties. As our meta-atoms exhibit mirror-reflection symmetries on the *xoy* plane with two principal axes denoted by $\hat{u}$ and $\hat{v}$ , respectively, we can capture their reflection Jones Matrices as $R=\left(\begin{array}{cc} r_{uu} & 0\\ 0 & r_{vv} \end{array}\right)$ in linear-polarization bases, with *r*_*uu*_ and *r*_*vv*_ denoting two complex reflection coefficients for two linear polarizations. Shining a particular meta-atom with a CP light, our previous studies [28], [29] have shown that the reflected beam normally contains two modes – a spin-preserved normal mode with a complex amplitude $\left(r_{uu}+r_{vv}\right)/2$ and a spin-flipped anomalous mode with a complex amplitude $\left(r_{uu}-r_{vv}\right){\text{e}}^{\text{i}\sigma 2\xi }/2$ , where $\left|\sigma \right\rangle =\left|+\right\rangle$ and $\left|-\right\rangle$ denote the LCP and RCP states, respectively. We note that the anomalous mode carries an extra spin-dependent phase *σ* ⋅ Φ_Geo_ with Φ_Geo_ = 2*ξ*, which is highly desired for further wave manipulation. To achieve a highest possible working efficiency, we need to suppress the undesired normal mode yielding the following condition

(1) $$A_{n}=\left|r_{uu}+r_{vv}\right|/2=0$$

**Figure 2:**
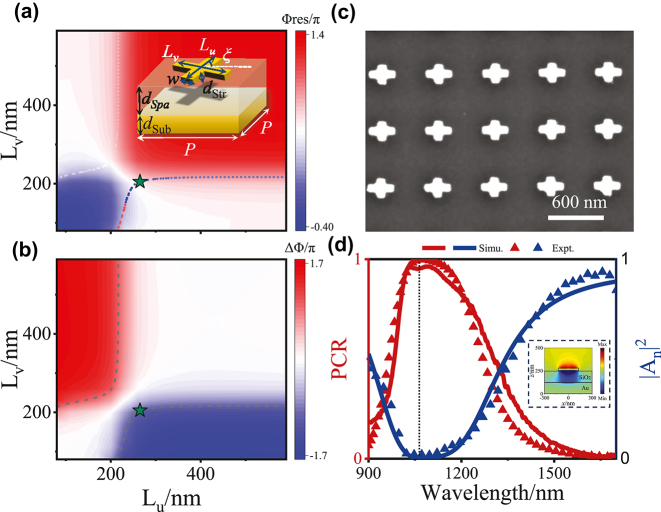
Phase diagrams and experimental characterizations of a representative meta-atom. (a) Resonance phase Φ_Res_ and (b) phase difference ΔΦ as functions of *L*_*u*_ and *L*_*v*_, calculated by FDTD simulations at the wavelength of 1,064 nm, with dashed lines indicating ΔΦ = ±*π*. Inset schematically illustrates the geometry of a typical meta-atom employed in this work. (c) SEM image of a fabricated sample containing an array of meta-atoms with structural parameters *L*_*u*_ = 265 nm and *L*_*v*_ = 205 nm, marked by a green star in (a) and (b). (d) Spectra of *PCR* and ${\left|A_{n}\right|}^{2}$ obtained by simulations (lines) and measurements (triangles) for the fabricated metasurface. Inset shows the FDTD-simulated Re(*H*_*y*_) field distributions on the *x*–*z* plane intersecting the metallic structure at the resonant frequency, as the meta-atom is shined by LP light with **E** field oriented along the *u*-axis.

Under such a condition, wave reflected by the meta-atom can be rewritten as

(2) $$R\left|\sigma \right\rangle =A_{a}{\text{e}}^{\text{i}\left(\Phi_{\text{Str}}+\sigma \cdot \Phi_{\text{Geo}}\right)}\left|-\sigma \right\rangle$$

where $\Phi_{\text{Str}}=\arg\left(r_{uu}-r_{vv}\right)$ denotes the structural phase and

(3) $$A_{a}=\left|r_{uu}-r_{vv}\right|/2$$

is the strength of the anomalous mode. Clearly, the reflection phase of such a meta-atom contains both a spin-*independent* structural phase Φ_Str_ and a spin-*dependent* geometric phase *σ* ⋅ Φ_Geo_, thus, the reflection phases under distinct circular-polarization incidences can be separately designed, which is crucial to realize bi-functional controls over CP lights. We note the resonant modes supported by our MIM meta-atoms are lateral resonances (see the inset to Figure 2(d) for simulated field distribution of a typical meta-atom at resonance) rather than the Fabry–Perot vertical resonances in dielectric resonators [30]. As such, our meta-atoms can exhibit deep-subwavelength thicknesses (*d*_spa_ ≪ *λ*), in sharp contrast to the wavelength-scale-high dielectric resonators frequently adopted in constructing bi-functional meta-holograms previously. Employing composite MIM meta-atoms as our building blocks, the constructed bi-functional meta-devices exhibit both high efficiencies and ultra-thin thicknesses, showcasing advantages over previous ones utilizing dielectric structures.

We then explicitly illustrate how to design appropriate meta-atoms with desired reflection phases. As shown in the inset to Figure 2c, we employ silicon dioxide (SiO_2_) as the spacer, and optimize its thickness as *d*_Spa_ = 125 nm via full-wave simulations to ensure that our meta-atoms are in the under-damped regime exhibiting nearly 2*π* phase coverage as the frequency varies and possess very low absorption [31]. The thickness of the continuous gold (Au) film is set as *d*_sub_ = 125 nm to minimize light transmission, and the periodicity based on which we fabricate the meta-atom array is set as *P* = 600 nm. The top resonator is an Au cross composed of two bars with lengths *L*_*u*_ and *L*_*v*_, respectively, the widths of which are set as *w* = 80 nm. Neglecting losses, we can write the reflection coefficients of our meta-atoms (arranged in periodic arrays) as $r_{uu}={\text{e}}^{\text{i}\Phi_{u}}$ and $r_{vv}={\text{e}}^{\text{i}\Phi_{v}}$ , respectively. Therefore, we can re-write Eq. (1) as

(4) $$\Delta \Phi =\Phi_{v}-\Phi_{u}=\pm \pi ,$$

indicating that the meta-atoms should behave as perfect half-wave-plates (HWPs). Meanwhile, the structural phase can also be explicitly derived as

(5) $$\Phi_{\text{Res}}=\left(\Phi_{u}+\Phi_{v}\right)/2-\pi /4$$

under the condition Eq. (1) or Eq. (4). Therefore, all properties of the meta-atoms can be unambiguously determined by their reflection phases Φ_*u*_ and Φ_*v*_, which in turn, can be efficiently tuned by changing two structural parameters *L*_*u*_ and *L*_*v*_.

Fixing the working wavelength at 1,064 nm, we show in Figure 2a and b how the FDTD-calculated Φ_*Res*_ and ΔΦ of our meta-atom vary against *L*_*u*_ and *L*_*v*_ (see Figure S1 in SI for the Φ_*u*_ and Φ_*v*_ phase diagrams), respectively. Two dashed lines in Figure 2a and b represent the ΔΦ = ±*π* lines where Eq. (1) and Eq. (4) are satisfied, implying that we can only choose meta-atoms with structural parameters sitting on these two lines. Meanwhile, we find from Figure 2a that meta-atoms sitting on these two lines can still exhibit Φ_Str_ varying in a wide range, providing us a large parameter space to choose the suitable meta-atoms from.

As an example, we experimentally characterize the optical properties of a typical meta-atom selected from the ΔΦ = ±*π* lines, denoted by a green star in Figure 2a and b. We fabricate a metasurface sample consisting of a periodic array of meta-atoms with given structural parameters using standard electron-beam lithography (EBL) technology (see Section 4 for fabrication details). Figure 2c demonstrates a scanning-electron-microscope (SEM) image of the fabricated sample. We then experimentally measure the reflectance spectra ( $R_{u}={\left|r_{u}\right|}^{2}$ and $R_{v}={\left|r_{v}\right|}^{2}$ ) of the sample under illuminations of linearly polarized (LP) lights with **E** fields oriented along *u*- and *v*-axes, respectively (see Section 2 of SI). Unfortunately, our experimental setup does not allow us to directly measure the reflection-phase spectra. Alternatively, we measure the spectrum for the polarization conversion ratio (*PCR*) of the sample, which is defined as the ratio between the power of the cross-polarized reflection signal and that of the incident LP light with **E** field oriented along the 45° angle between $\hat{u}$ and $\hat{v}$ axes. We note that the PCR thus defined is closely related to the reflection-phase difference ΔΦ of the sample, which can be expressed as

(6) $$PCR=\frac{1}{2}\left(1-2\frac{\sqrt{R_{u}R_{v}}\cos\Delta \Phi }{R_{u}+R_{v}}\right),$$

where a 100 % *PCR* corresponds precisely to the case of ΔΦ = ±*π* as desired. Moreover, the normal-mode strength *A*_*n*_ can be retrieved from the above raw experimental data, with:

(7) $$A_{n}=\sqrt{\left(R_{u}+R_{v}\right)\left(1-PCR\right)/2}.$$

Figure 2d depicts the measured spectra of *PCR* and ${\left|A_{n}\right|}^{2}$ of our sample, which are in excellent agreement with the corresponding simulation results. We find that *PCR* ≈ 1 (and ${\left|A_{n}\right|}^{2}\approx 0$ consistently) at the working wavelength 1,064 nm, indicating that the meta-atom indeed behaves as a nearly-perfect HWP as desired. Other meta-atoms can be experimentally characterized in a similar way. Based on such a meta-atom database, we are now ready to design any multi-functional metasurface once its phase distributions are known.

### 2.2 Benchmark test: a single-functional meta-hologram

**Figure 3:**
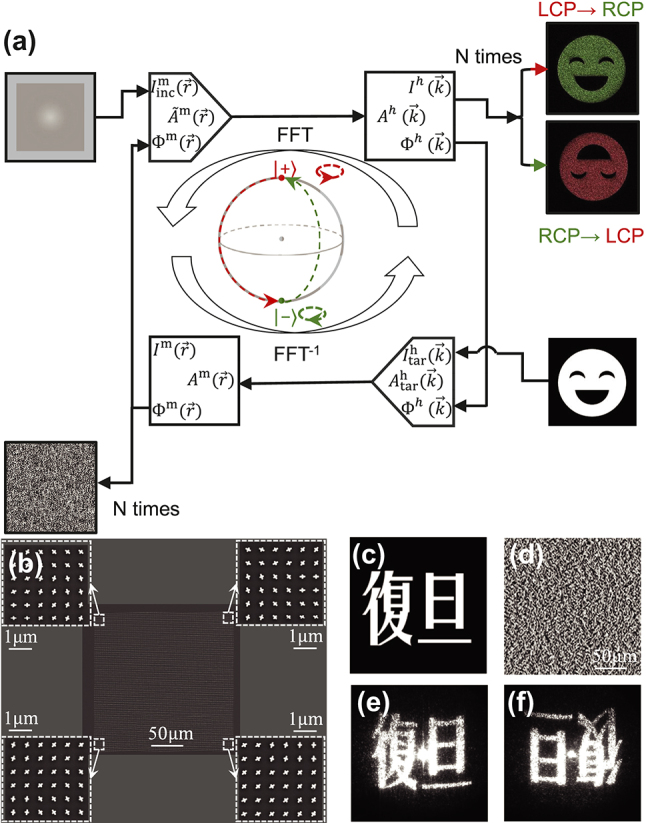
Single-functional hologram based on metasurface. (a) Flow chart of the GS algorithm for designing a single-functional meta-hologram. (b) SEM image of the fabricated meta-hologram, with the insets on four corners depicting zoomed-in SEM images. (c) Target holographic image. (d) Phase distribution of the metasurface retrieved from the target image assuming that the incident light is an LCP beam at 1,064 nm. Experimentally recorded holographic images generated by the meta-hologram under (e) LCP incidence and (f) RCP incidence, respectively.

As a benchmark test of our generic approach, we first experimentally demonstrate a single-functional high-efficiency meta-hologram. In this case, we only need to retrieve the reflection phase distribution $\Phi_{\text{tar}}^{m}\left(\vec{r}\right)$ of our metasurface from a single target holographic image with FF intensity distribution given as $I_{\text{tar}}^{h}\left(\vec{k}\right)$ . The retrieval process is schematically shown in Figure 3(a). Starting from $A_{\text{tar}}^{h}\left(\vec{k}\right)=\sqrt{I_{\text{tar}}^{h}\left(\vec{k}\right)}{\text{e}}^{\text{i}\Phi_{\text{Ran}}^{h}\left(\vec{k}\right)}$ describing the FF amplitude distribution of the holographic image with $\Phi_{\text{Ran}}^{h}\left(\vec{k}\right)$ being an initial random phase distribution, we perform inverse Fourier-transformation to obtain the near-field (NF) amplitude distribution $A^{m}\left(\vec{r}\right)=\sqrt{I^{m}\left(\vec{r}\right)}{\text{e}}^{\text{i}\Phi^{m}\left(\vec{r}\right)}$ on the metasurface plane. Next, we replace the retrieved intensity distribution $I^{m}\left(\vec{r}\right)$ by that of the incident light $I_{\text{inc}}^{m}\left(\vec{r}\right)$ to form a new NF distribution ${\tilde{A}}^{m}\left(\vec{r}\right)=\sqrt{I_{\text{inc}}^{m}\left(\vec{r}\right)}{\text{e}}^{\text{i}\Phi^{m}\left(\vec{r}\right)}$ , and then perform Fourier transformation to get the FF amplitude distribution $A^{h}\left(\vec{k}\right)=\sqrt{I^{h}\left(\vec{k}\right)}{\text{e}}^{\text{i}\Phi^{h}\left(\vec{k}\right)}$ . In the last step of a single iteration loop, we use the target intensity distribution $I_{\text{tar}}^{h}\left(\vec{k}\right)$ to replace the retrieved one $I^{h}\left(\vec{k}\right)$ , and obtain a new FF amplitude distribution $A_{\text{tar}}^{h}\left(\vec{k}\right)=\sqrt{I_{\text{tar}}^{h}\left(\vec{k}\right)}{\text{e}}^{\text{i}\Phi^{h}\left(\vec{k}\right)}$ . Such iterations are repeated until the difference between $\Phi^{m}\left(\vec{r}\right)$ calculated in two adjacent steps is less than a threshold, and then we get the converged NF distribution on the metasurface $\Phi_{\text{tar}}^{m}\left(\vec{r}\right)$ . In our case, we typically set the iteration number as *N* = 400 to get the convergent results.

We now design a metasurface that, under the LCP incidence at 1,064 nm, can generate the target FF holographic image (two Chinese characters meaning “Fudan”) shown in Figure 3(c). We first follow the procedures describe above to retrieve the required phase distribution $\Phi_{\text{tar}}^{m}\left(\vec{r}\right)$ of the metasurface, and depict it in Figure 3(d). As our metasurface is only required to generate the correct holographic image under LCP light incidence, we can simply use the PB mechanism to construct our metasurface. Employing the meta-atom experimentally demonstrated in Figure 2(d) as the building block, we use the formula $\xi \left(\vec{r}\right)=\Phi_{\text{tar}}^{m}\left(\vec{r}\right)/2$ to determine the rotation angles of meta-atoms located at different positions, and then fabricate a sample according to the rotation-angle distribution. Figure 3(b) depicts the SEM image of our fabricated sample, with the insets showing 4 zoomed-in pictures of the corner areas.

We now experimentally characterize the fabricated meta-hologram. Figure 3(f) illustrates the experimentally recorded pattern on the imaging plane (see SI for our optical characterization setup), as the metasurface is shined by an LCP light at 1,064 nm. The recorded image matches well with the pre-designed pattern as shown in Figure 3(c). For completeness, we also experimentally recorded the field pattern obtained on the same imaging plane as the metasurface is shined by an RCP light at 1,064 nm. The PB mechanism ensures that our metasurface should exhibit the phase distribution $-\Phi_{\text{tar}}^{m}\left(\vec{r}\right)$ under RCP light incident. Thus, after Fourier transformation, we can prove that the FF pattern under the RCP incidence should be an exact centro-symmetric pattern of that obtained under the LCP incidence, which is indeed the case as shown in Figure 3(f).

### 2.3 Bi-functional meta-holograms

We now experimentally demonstrate a series of metasurfaces that can generate two distinct pre-designed holographic images, as shined by LCP and RCP light beams, respectively. We use a parallel GS algorithm to retrieve two reflection-phase distributions ( $\Phi_{\text{tar},\left|+\right\rangle }^{m}\left(\vec{r}\right)$ and $\Phi_{\text{tar},\left|-\right\rangle }^{m}\left(\vec{r}\right)$ ) exhibited by the metasurfaces under the illuminations of CP light beams with different spins $\left|\sigma \right\rangle =\left|\pm \right\rangle$ , from two target holographic images described by FF distributions $I_{\text{tar},\left|\sigma \right\rangle }^{h}\left(\vec{k}\right)$ . The calculation procedures are essentially the same as that described in the last section, with the only difference being that here we need to retrieve two phase functions independently.

With $\Phi_{\text{tar},\left|+\right\rangle }^{m}\left(\vec{r}\right)$ and $\Phi_{\text{tar},\left|-\right\rangle }^{m}\left(\vec{r}\right)$ retrieved, we next sort out a series of meta-atoms with appropriate spin-dependent reflection phases. According to Eq. (2), we find that

(8) $$\Phi_{\text{tar,}\left|\sigma \right\rangle }^{m}\left(\vec{r}\right)=\Phi_{\text{Str}}\left(\vec{r}\right)+\sigma \Phi_{\text{Geo}}\left(\vec{r}\right),$$

where $\Phi_{\text{Str}}\left(\vec{r}\right)\;\text{and}\;\sigma \Phi_{\text{Geo}}\left(\vec{r}\right)$ represent, respectively, the structural and geometric phases of the meta-atom located at position $\vec{r}$ . Furthermore, since our meta-atoms are required as HWPs to suppress undesired normal-mode scatterings and obtain $\Phi_{\text{Geo}}\left(\vec{r}\right)=2\xi \left(\vec{r}\right)$ , we derive from Eq. (8) that

(9) $$\left\{\begin{array}{c} \Phi_{\text{Str}}\left(\vec{r}\right)=\frac{\Phi_{\text{tar},\left|+\right\rangle }^{m}\left(\vec{r}\right)+\Phi_{\text{tar},\left|-\right\rangle }^{m}\left(\vec{r}\right)}{2}\\ \Delta \Phi \left(\vec{r}\right)=\pm \pi \\ \xi \left(\vec{r}\right)=\frac{\Phi_{\text{tar},\left|+\right\rangle }^{m}\left(\vec{r}\right)-\Phi_{\text{tar},\left|-\right\rangle }^{m}\left(\vec{r}\right)}{4} \end{array}\right.$$

which can guide us to search for appropriate meta-atoms at different positions. Specifically, the first two equations can help us determine from Figure 2(a) and (b) the structural parameters *L*_*u*_ and *L*_*v*_ of our meta-atoms, while the third equation tells us the rotation angles of all meta-atoms.

**Figure 4:**
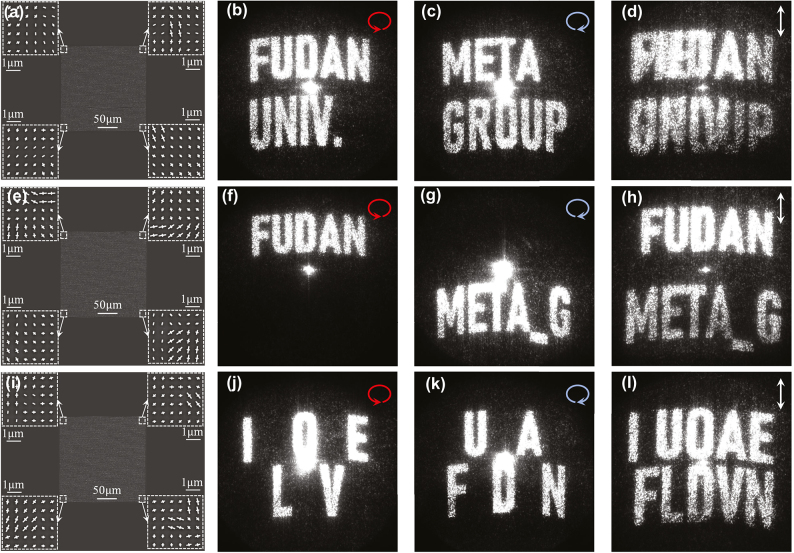
Bi-functional hologram based on metasurfaces. (a) SEM image of a full-screen bi-functional meta-hologram, and measured holographic images generated by it under (b) LCP, (c) RCP, and (d) LP incidences, respectively. (e) SEM image of a split-screen bi-functional meta-hologram, and measured holographic images generated by it under (f) LCP, (g) RCP and (h) LP incidences, respectively. (i) SEM image of an information-encrypted meta-hologram, and measured holographic images generated by it under (j) LCP, (k) RCP and (l) LP incidences, respectively.

Following the above-mentioned procedures, we design and fabricate three different metasurfaces that can realize three distinct bi-functional holographic images. With these samples at hand, we next utilize the same experimental setup as in the last section to characterize the performances of these devices. Shine three metasurfaces by normally incident light beams at 1,064 nm with polarizations LCP, RCP and LP, respectively, we obtain the corresponding images recorded on the holographic plane of our system, and then demonstrate them in the last three rows of Figure 4. Taking the first metadevice as an example, we find from Figure 4(b) and (c) that two different patterns (i.e., “FUDAN UNIV.” and “META GROUP”) are displayed clearly on the imaging plane, in the cases of LCP and RCP incidences, respectively. As the incident polarization changes to LP containing both LCP and RCP components, we find that the generated holographic image contains two pre-designed patterns simultaneously (see Figure 4(d)). The other two meta-holograms exhibit similar bi-functionalities with only the pre-designed target patterns changed accordingly, as experimentally demonstrated in Figure 4(f)–(h) and (j)–(l), respectively. In particular, we note that the third metadevice can be applied to information encryption. Carefully arranging the two groups of desired character patterns, we find that while effective information (e.g., “I LOVE” and “FUDAN”) can be correctly displayed as the device is shined by LCP and RCP light beams (see Figure 4(j) and (k)), respectively, no meaningful information can be obtained as the incident light changes to the LP beam (see Figure 4(l)).

Finally, we employ full-wave simulations and experimental measurements to estimate the working efficiencies of our bi-functional meta-holography. In our design, only the abnormal components carrying the designated phases contribute to generating the desired patterns, thus the efficiency of our metaatom is defined as $\eta ={\left|A_{a}\right|}^{2}$ . As a result, the overall efficiency of our device is determined by the average light strength carried by these abnormal components scattered by our meta-atoms. Detailed analyses and comparisons among different schemes are presented in Section 3 of SI. The numerically evaluated efficiencies of our bi-functional meta-holograms are around 48 %, while the experimentally evaluated efficiencies reach approximately 35 %, which are mainly limited by inevitable metallic absorptions in this frequency domain. Comparisons with other schemes proposed previously clearly highlight the key advantage of our strategy, which can realize bi-functional holography well balancing the requirements on high working efficiency and ultra-thin device thickness.

## 3 Conclusions

To conclude, we proposed a generic strategy to realize bi-functional meta-holograms and experimentally verify the concept in the telecom frequency domain. Compared with other schemes developed previously, our bi-functional meta-holograms can well balance the requirements on high efficiency and ultra-thin device thickness. After designing a series of meta-atoms exhibiting incident-spin-delinked reflection phases and experimentally characterize one typical meta-atom, we experimental realize a single-functional meta-hologram as a benchmark test. We next employ the designed meta-atoms to construct a series of metasurfaces and experimentally demonstrate that they can realize two distinct holographic images, as shined by CP light at 1,064 nm with different helicities. The proposed meta-platform can find many applications in integration photonics, such as information encryption, anticounterfeiting, data storage, etc.

## 4 Methods

### 4.1 Numerical simulation

In our finite-difference time-domain simulations, the permittivity of Au is described by the Drude model $\varepsilon_{r}\left(\omega \right)=\varepsilon_{\infty }-\frac{\omega_{p}^{2}}{\omega \left(\omega +i\gamma \right)}$ , with *ɛ*_*∞*_ = 9, *ω*_*p*_ = 1.367 × 10^16^ s^−1^, *γ* = 2.448 × 10^14^ s^−1^, obtained by fitting with experimental results. The SiO_2_ spacer is considered as a lossless dielectric with permittivity *ɛ* = 2.25. Additional losses caused by surface roughness and grain boundary effects in thin films as well as dielectric losses are effectively considered in the fitting parameter *γ*.

### 4.2 Sample fabrications

All MIM samples are fabricated using standard thin-film deposition and EBL techniques. In the first step, we sequentially deposite 5 nm – thick Cr, 125 nm – thick Au, 5 nm Cr and a 125 nm – thick SiO_2_ dielectric layer onto a silicon substrate using magnetron DC sputtering (Cr and Au) and RF sputtering (SiO_2_). Then, we lithograph the cross structures with EBL, employing an ∼100 nm thick PMMA2 layer at an acceleration voltage of 20 keV. After development in a solution of methyl isobutyl ketone and isopropyl alcohol, a 5 nm Cr adhesion layer and a 30 nm Au layer are subsequently deposited using thermal evaporation. The Au patterns are finally formed on top of the SiO_2_ film after a lift-off process using acetone.

### 4.3 Experimental setup

We use a near-infrared (NIR) microimaging system to characterize the performance of all designed meta-atoms. A broadband supercontinuum laser (Fianium SC400) source and a fibre-coupled grating spectrometer (Ideaoptics NIR2500) are used in the FF measurements. A beam splitter, a linear polarizer and a CCD are also used to measure the reflectance and analyze the polarization distributions.

## Supplementary Material

Supplementary Material Details

## List of abbreviations

CP: circular-polarized
DOFs: degrees of freedom
EP: elliptical-polarized
FF: far-field
GS: Gerchberg–Saxton
LCP: left circular polarization
LP: linear-polarized
MIM: metal-insulator-metal
NIR: near-infrared
PB: Pancharatnam–Berry
RCP: right circular polarization
SEM: scanning electron microscopy
