# Supplementary material for: High-efficiency generation of bi-functional holography with metasurfaces
Source: Nanophotonics. 2025 Feb 20;14(8):1283–90. doi: 10.1515/nanoph-2024-0677 (PMC12019932; doi:10.1515/nanoph-2024-0677)
Supplement: Supplementary file 1 — Supplementary Material Details [file j_nanoph-2024-0677_suppl_001.docx]

Supplementary Information for

**High-efficiency generation of bi-functional holography with metasurfaces**

Changhong Dai^1†^, Tong Liu^2†^, Dongyi Wang^3*^, Lei Zhou^2,4*^

^1.^ State Key Laboratory of Surface Physics, Key Laboratory of Micro and Nano Photonic Structures (Ministry of Education), Shanghai Key Laboratory of Metasurfaces for Light Manipulation and Department of Physics,
Fudan University, Shanghai 200438, China

^2.^ Department of Physics, The Hong Kong University of Science and Technology, Clear Water Bay, Kowloon, Hong Kong, China

^3.^ Department of Physics, University of Hong Kong, Hong Kong, China

^4.^  Shanghai Key Laboratory of Metasurfaces for Light Manipulation
Shanghai, 200433, China

^*^Corresponding Authors: Dongyi Wang, Email: [physwang@hku.hk](mailto:physwang@hku.hk)

Lei Zhou, E-mail: [phzhou@fudan.edu.cn](mailto:phzhou@fudan.edu.cn)

†These authors contributed equally to this work

**Section 1. Simulation results on MIM meta-atoms**


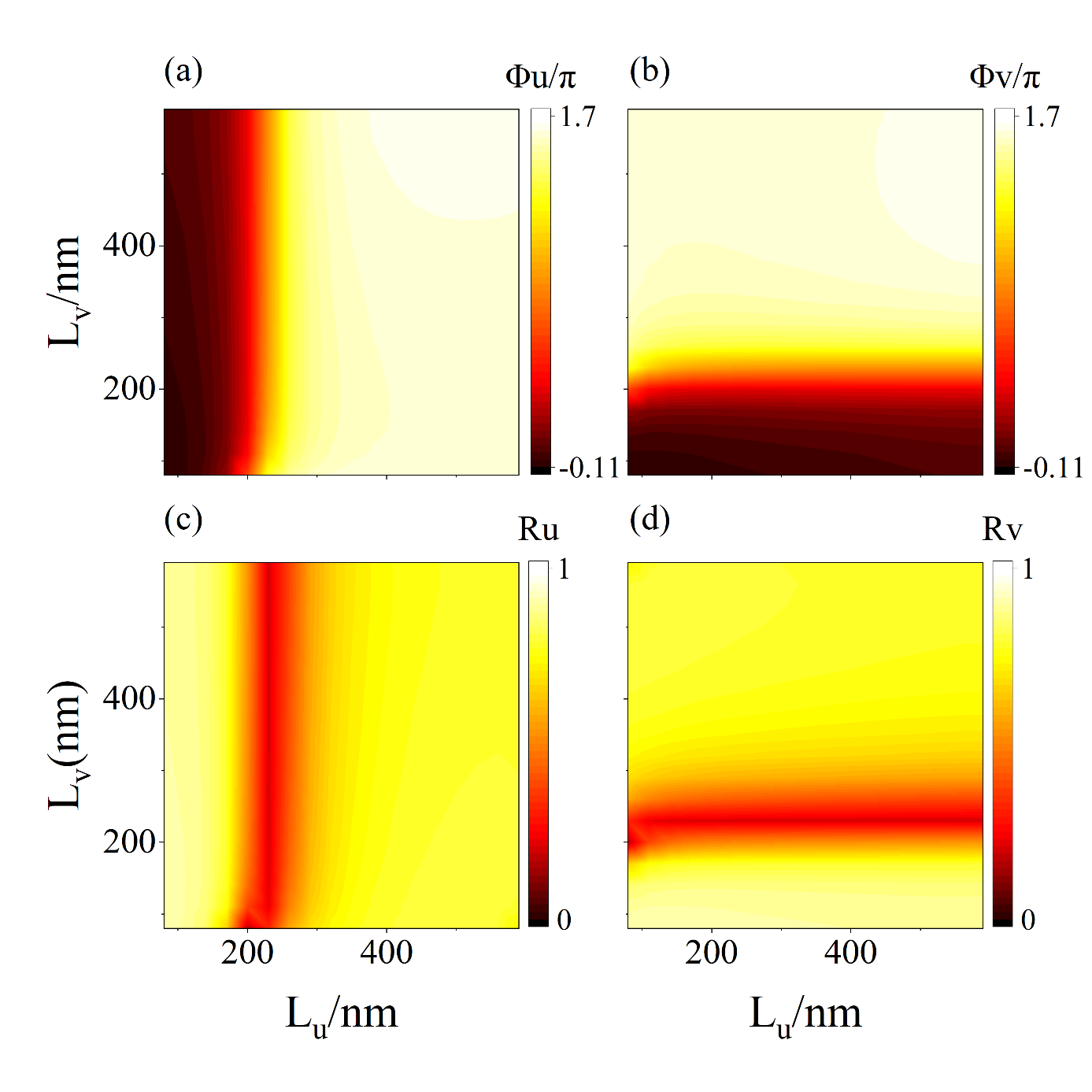


Fig. S1 FDTD-simulated reflectance (a) $\text{R}_{\text{u}}\text{=}{\text{|}\text{r}_{\text{uu}}\text{|}}^{\text{2}}$ and (b) $\text{R}_{\text{v}}\text{=}{\text{|}\text{r}_{\text{vv}}\text{|}}^{\text{2}}$, as well as reflection phases (c) $\text{Φ}_{\text{u}}$ and (d) $\text{Φ}_{\text{v}}$, with respect to the variations in $\text{L}_{\text{u}}$ and $\text{L}_{\text{v}}$ for MIM meta-atoms, under illuminations of $\hat{\text{u}}$-oriented and $\hat{\text{v}}$-oriented LP light, respectively. The working wavelength is 1064 nm.

**Section 2. Supplementary results for the meta-atom studied in Fig. 2**

To experimentally characterize the optical properties, we first measure the reflection spectra of the sample, which features the corresponding meta-atoms arranged periodically, under the illuminations of linearly polarized (LP) light with **E** field polarized along the u- and v-axis, respectively. The measured results are shown in Figs. S2(a), alongside their simulated counterparts for comparison. We next shine the sample by LP light with **E** field oriented at an angle of 45° with respect to the u-axis and measure the normalized power patterns at the working wavelength of 1064 nm to evaluate the polarization-conversion capabilities of these meta-atoms. As shown in Figs. S2(b), the measured and simulated power patterns indicate that the meta-atom functions as a half-wave plate with $\text{∆}\text{Φ}\text{ }\text{=}\text{ }\text{π}$.


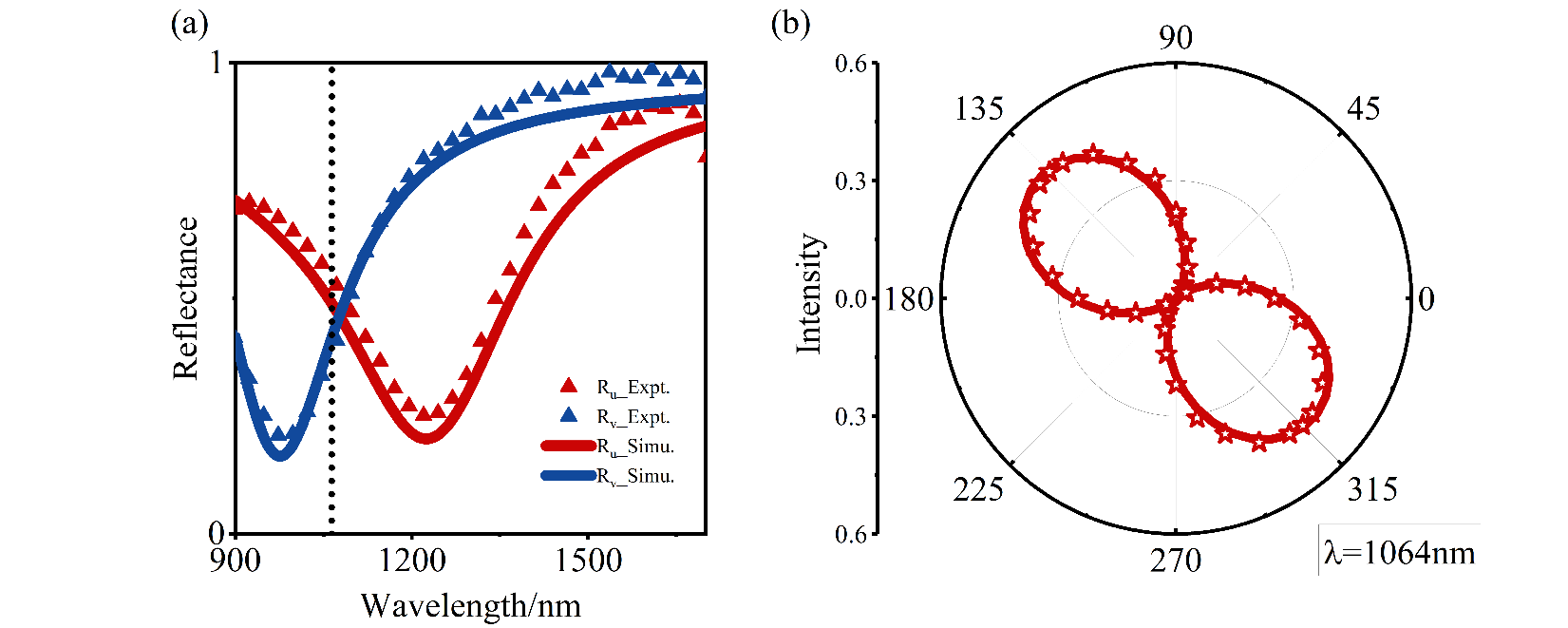


Fig. S2 Experimentally tested (circle) and FDTD-simulated (line) reflectance spectrum (a) of the meta-HWP along *u*, *v-* axes, with the dashed line indicating centre working wavelength (𝛌= 1064 nm); and corresponding intensity pattern filtered by a polarizer tilted at different angles to filter different components of the reflected light (b) at 45°polarized LP incidence. The incident 45° LP polarized light is converted to 135° LP polarized ones for the meta-atom, being a solid proof of performant half-wave-plates.

**Section 3. Comparative advantages of our work: enhanced efficiency together with ultra-thin design**

In this section, we highlight the advantages of our work, which is characterized by simultaneous realization of both higher efficiency and ultra-thin design compared to some typical previous studies.

We first investigate the efficiency. Numerical calculations are conducted to evaluate the efficiency of the delinked bi-functional holography generators. As illustrated in the manuscript, $\text{A}_{\text{a}}$ represents the intensity ratio of the desired component in the reflected wave, which is intrinsically linked to the efficiency of each meta-atom, expressed as

*η*$\text{ =}{\text{ }\left| \text{A}_{\text{a}} \right|}^{\text{2}}$ (S1)

By calculating the efficiency of each meta-atom using Eq. (S1) and the average value, the working efficiencies of delinked bi-functional holography generators can be determined. Following this approach, the average efficiency of the delinked bi-functional holography generators is evaluated to be $\bar{\text{η}}\text{ }\text{=}\text{ }\text{48.08 \%}$.

We next illustrate how to evaluate the experimental working efficiency of the delinked bi-functional holography generators. Take the full-screen bi-functional meta-hologram as an example, we first collect two holographic patterns on the image plane for LCP and RCP incidences, respectively [Fig. 4b and 4c]. Due to inherent metallic losses, the reflection amplitudes of the meta-atoms are less than 1 in the real design, which means that the condition for zero normal mode strength cannot be strictly met. As a result, some normal mode components remain, which are focused by the imaging lens onto the imaging plane, forming a central light spot. Removing the central light spot, we can directly sum the intensities of the remaining pixels in both holographic images to obtain the power $P_{LCP}$ and $P_{RCP}$ respectively. We then evaluate the incident power. Shine the same Gaussian beam onto a 125-nm-thick silver film, we experimentally measure the incident power $P_{inc}$ through integration. Given that the focused spot is a spherical wave, the intensity of the spot decays exponentially, following a Gaussian distribution along $x$ and $y$ directions, as described by:

$I\left( x,y \right)=I_{m}e^{-\frac{1}{2}\left( \frac{x-x_{0}}{\sigma_{x}} \right)^{2}-\frac{1}{2}\left( \frac{y-y_{0}}{\sigma_{y}} \right)^{2}}$ (S2)

where $I_{m}$ is the peak strength, $x_{0}$ and $y_{0}$ are the coordinates of the spot centre, and $\sigma_{x}$, $\sigma_{y}$ are the standard deviations of $x$ and $y$ orientations, respectively. To calculate the incident power, we perform the following integration:

$P_{inc}=\int_{-\infty}^{+\infty} \int_{-\infty}^{+\infty} I_{m}e^{-\frac{1}{2}\left( \frac{x-x_{0}}{\sigma_{x}} \right)^{2}-\frac{1}{2}\left( \frac{y-y_{0}}{\sigma_{y}} \right)^{2}}dxdy=2\pi I_{m}\sigma_{x}\sigma_{y}$ (S3)

Finally, we can evaluate the bi-functional holography working efficiency as

$C=\frac{P_{LCP}+P_{RCP}}{2P_{inc}}$ (S4)

Employing this calculation procedure, the experimental efficiencies of three bi-functional holographic meta-devices presented in the main text are evaluated to be 36.8%, 32% and 34.2% respectively, with their counterpart for the single-functional holographic meta-device reaching 38%.

|  | Our work | [1] | [2] | [3] | [4] | [5] | [6] | [7] |
| --- | --- | --- | --- | --- | --- | --- | --- | --- |
| Efficiency | >30% | <20% | <20% | >30% | >30% | <20% | <20% | <20% |
| Thickness | ~1/4 $\text{λ}$ | $\text{≫}\text{ }\text{λ}$ | ~1/3$\text{ λ}$ | ~$\text{ λ}$ | $\text{\textasciitilde5 λ}$ | ~$\text{1/2 λ}$ | $\text{\textasciitilde2 λ}$ | ~$\text{1/2 λ}$ |

Tab. S1 Comparison of bi-functional holography generators proposed in our work and their counterparts from previous references [1-7]

Here, we also investigate previous works on linear polarization multiplexing meta-hologram[1], polarization-controlled dual-hologram generation meta-device[2], chiral-hologram generation meta-device[3], controllable multiwavelength meta-hologram[4], vectorial holograms with spatially continuous polarization distributions[5], vectorial Fourier metasurfaces[6] and dynamic vectorial holography[7] for comparison, as shown in Table S1. The holography generators proposed in this work are distinguished by the simultaneous realization of both significantly high efficiency (>30%) and ultra-thin design (~1/4 $\text{λ}$). The enhanced efficiency of our work stems from the effective design strategy which utilizes phases governed by both structural resonance and geometric rotation while suppressing the undesired normal modes, with material loss being the sole contributor to any imperfection in efficiency. As noted in the main text, the ultra-thin design originates from lateral resonance[8], enabling our MIM system to attain ultra-thin thickness (subwavelength scale), while previous endeavours employing propagating phases, though also performant in efficiency, still suffer from a relatively thick profile, thus can’t simultaneous access both high efficiency and ultra-thinness.

**Section 4. Schematics of our experimental setups**


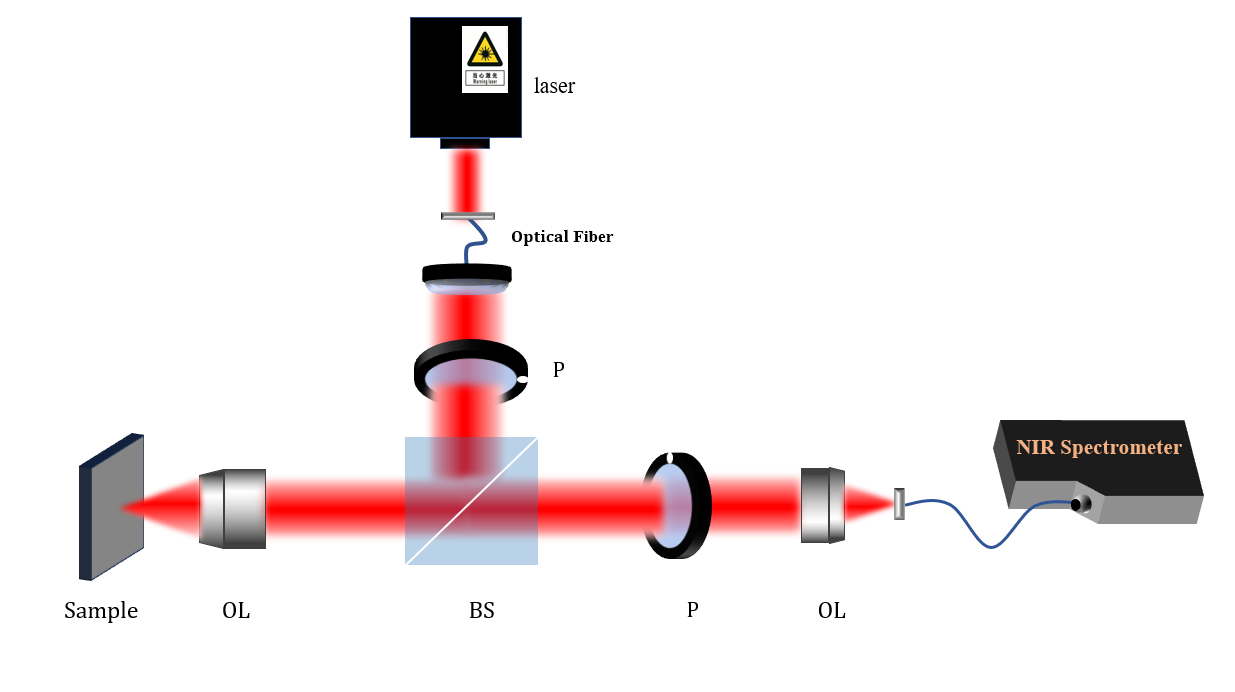


Fig. S3 Schematics of the experimental setup for meta-half-wave plate characterization. P represents linear Polarizer, OL represents Objective Lens, and BS represents Beam Splitting lens.


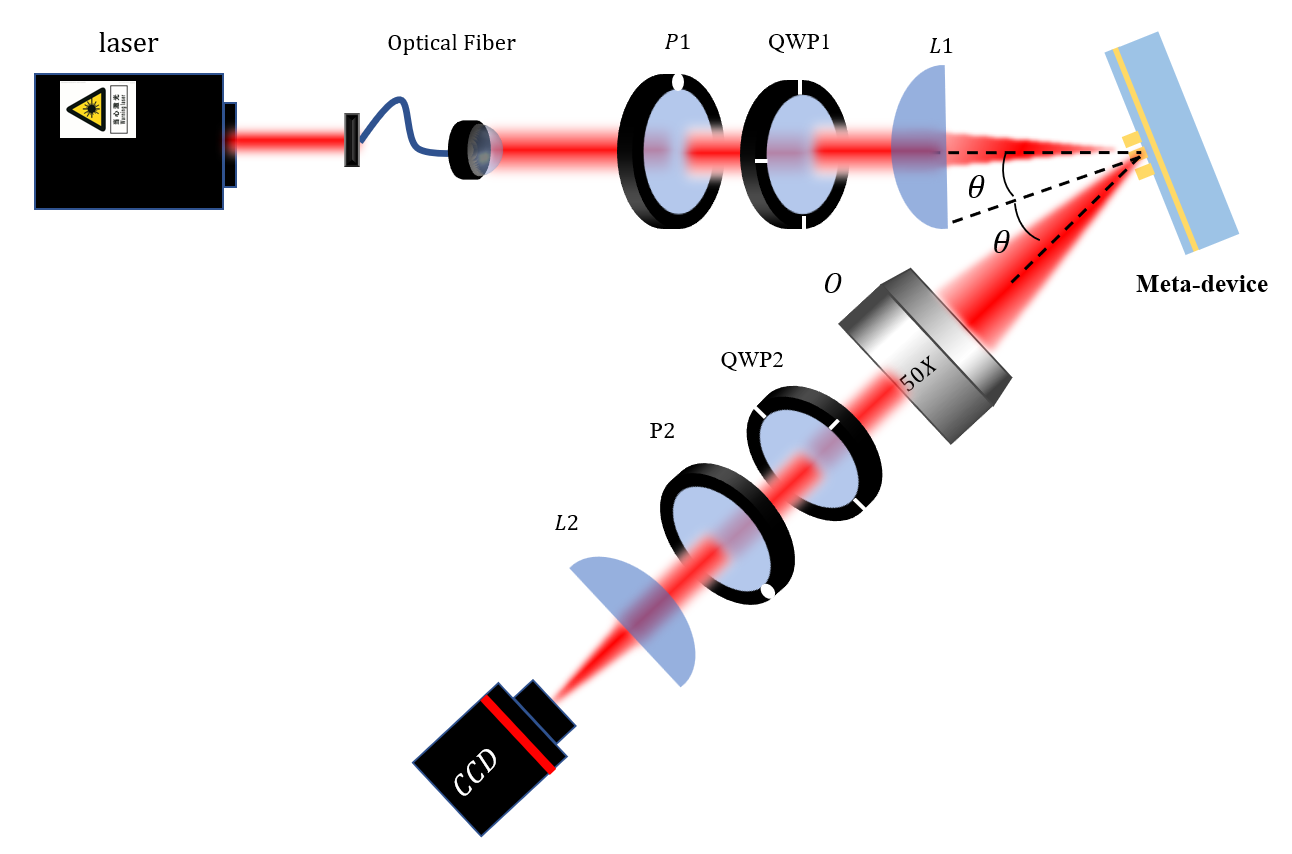


Fig. S4 The micro-imaging system for far-field holography characterization.

A micro-imaging system as shown in Fig. S4 is set up for far-field holography characterization. The polarization of the laser beam is converted to circular polarization through the combination of a polarizer P1 and a quarter-wave plate QWP1, which are oriented at a relative angle of 45°. Our meta-device is positioned at the focal plane of lens L1 (focal length being 175mm) with an incident angle of $\theta\approx22$°. A high numerical aperture (N.A.=0.55) condenser lens O, along with another lens L2 (focal length being 100mm), are employed to collect and focus the beam reflected by meta-holography generators. Filtering out the undesired normal component by a polarizer P2 and a quarter-wave plate QWP2, the far-field holographic pattern is recorded by a CCD camera placed at the focal plane of L2.

**References**

[1] Y. Montelongo, J. O. Tenorio-Pearl, W. I. Milne, et al., "Polarization switchable diffraction based on subwavelength plasmonic nanoantennas," *Nano Lett*, vol. 14, pp. 294-8, 2014.

[2] W. T. Chen, K. Y. Yang, C. M. Wang, et al., "High-efficiency broadband meta-hologram with polarization-controlled dual images," *Nano Lett.*, vol. 14, pp. 225-230, 2014.

[3] J. P. Balthasar Mueller, N. A. Rubin, R. C. Devlin, et al., "Metasurface Polarization Optics: Independent Phase Control of Arbitrary Orthogonal States of Polarization," *Phys. Rev. Lett.*, vol. 118, pp. 113901, 2017.

[4] Z. Shi, M. Khorasaninejad, Y.-W. Huang, et al., "Single-Layer Metasurface with Controllable Multiwavelength Functions," *Nano Lett.*, vol. 18, pp. 2420-2427, 2018.

[5] D. Wen, J. J. Cadusch, J. Meng, et al., "Vectorial Holograms with Spatially Continuous Polarization Distributions," *Nano Lett*, vol. 21, pp. 1735-1741, 2021.

[6] Q. Song, A. Baroni, P. C. Wu, et al., "Broadband decoupling of intensity and polarization with vectorial Fourier metasurfaces," *Nat Commun*, vol. 12, pp. 3631, 2021.

[7] S. Zhang, L. Huang, X. Li, et al., "Dynamic Display of Full-Stokes Vectorial Holography Based on Metasurfaces," *ACS Photonics*, vol. 8, pp. 1746-1753, 2021.

[8] S. Ma, S. Xiao and L. Zhou, "Resonant modes in metal/insulator/metal metamaterials: An analytical study on near-field couplings," *Phys. Rev. B*, vol. 93, pp. 045305, 2016.
